# App-Based Salt Reduction Intervention in School Children and Their Families (AppSalt) in China: Protocol for a Mixed Methods Process Evaluation

**DOI:** 10.2196/19430

**Published:** 2021-02-10

**Authors:** Yuewen Sun, Rong Luo, Yuan Li, Feng J He, Monique Tan, Graham A MacGregor, Hueiming Liu, Puhong Zhang

**Affiliations:** 1 The George Institute for Global Health Peking University Health Science Center Beijing China; 2 Faculty of Medicine University of New South Wales Sydney Australia; 3 Barts and The London School of Medicine & Dentistry Queen Mary University of London London United Kingdom; 4 The George Institute for Global Health Sydney Australia

**Keywords:** mobile health, mobile phone, process evaluation, salt reduction, health education

## Abstract

**Background:**

The app-based salt reduction intervention program in school children and their families (AppSalt) is a multicomponent mobile health (mHealth) intervention program, which involves multiple stakeholders, including students, parents, teachers, school heads, and local health and education authorities. The complexity of the AppSalt program highlights the need for process evaluation to investigate how the implementation will be achieved at different sites.

**Objective:**

This paper presents a process evaluation protocol of the AppSalt program, which aims to monitor the implementation of the program, explain its causal mechanisms, and provide evidence for scaling up the program nationwide.

**Methods:**

A mixed methods approach will be used to collect data relating to five process evaluation dimensions: fidelity, dose delivered, dose received, reach, and context. Quantitative data, including app use logs, activity logs, and routine monitoring data, will be collected alongside the intervention process to evaluate the quantity and quality of intervention activities. The quantitative data will be summarized as medians, means, and proportions as appropriate. Qualitative data will be collected through semistructured interviews of purposely selected intervention participants and key stakeholders from local health and education authorities. The thematic analysis technique will be used for analyzing the qualitative data with the support of NVivo 12. The qualitative data will be triangulated with the quantitative data during the interpretation phase to explain the 5 process evaluation dimensions.

**Results:**

The intervention activities of the AppSalt program were initiated at 27 primary schools in three cities since October 2018. We have completed the 1-year intervention of this program. The quantitative data for this study, including app use log, activity logs, and the routine monitoring data, were collected and organized during the intervention process. After completing the intervention, we conducted semistructured interviews with 32 students, 32 parents, 9 teachers, 9 school heads, and 8 stakeholders from local health and education departments. Data analysis is currently underway.

**Conclusions:**

Using mHealth technology for salt reduction among primary school students is an innovation in China. The findings of this study will help researchers understand the implementation of the AppSalt program and similar mHealth interventions in real-world settings. Furthermore, this process evaluation will be informative for other researchers and policy makers interested in replicating the AppSalt program and designing their salt reduction intervention.

**International Registered Report Identifier (IRRID):**

DERR1-10.2196/19430

## Introduction

### Background

Dietary salt intake is a major risk factor for high blood pressure and cardiovascular diseases [1,2]. In 2017, 3 million deaths worldwide were attributable to excess salt intake [3]. Salt reduction is one of the *best-buys* recommended by the World Health Organization for the prevention and control of noncommunicable diseases (NCDs) [4]. Many countries have implemented their national strategies to reduce population salt intake through comprehensive strategies, including consumer education to raise awareness, food industry engagement to reformulate products, and front of package labeling to inform consumers’ choices [5]. Among these widely used strategies, reformulation is the most effective salt reduction strategy in high-income countries, where the majority of dietary salt intake comes in processed food [6]. For example, the United Kingdom, as the pioneer for salt reduction, achieved a 15% reduction in population salt intake between 2003 and 2011 by the gradual reformulation of prepackaged foods [7].

China is faced with a severe threat from NCDs, where more than 80% of the total disease burden is due to NCDs [8]. Salt intake in China is among the highest in the world [9]. The average salt intake of Chinese adults is about double the recommended maximum limits [9,10]. A study showed that approximately 80% of salt consumed in China is added by house cooks in daily cooking [11]. This dietary pattern makes it challenging to reduce salt intake because of the difficulties in changing individuals’ diet behaviors. To improve public awareness of high salt intake and its impacts, the Chinese government has initiated several national programs, such as the National Healthy Lifestyle Campaign [12]. However, an international study suggests that only 1.3% of the surveyed Chinese participants reported a reduced salt diet, which is much lower than that in Japan, the United States, and the United Kingdom [13]. Evidence indicates that sustained efforts are needed to effectively raise public awareness of salt and nudge behavior change in their daily lives.

People’s eating patterns and preferences are established early in life [14]. Hence, childhood is essential for building healthy dietary lifestyles to prevent related diseases, including cultivating low-salt diet habits. A salt reduction program in primary schools, named School-EduSalt, was conducted to investigate the potential benefits of salt reduction intervention among school children [15]. This one-term health education program on salt reduction among students achieved remarkable intervention outcomes and resulted in a 25% decrease in salt intake among adult family members [15]. This study shows that health education at schools can benefit not only school children but also their family members. This School-EduSalt program developed an intervention approach for salt reduction in the Chinese context with low cost [16]. However, the generalizability of this intervention model is limited because of its high requirements for health education teachers and the extra workload to schools.

With the development of modern technology during the past decades, mobile phones and apps have become a popular platform for lifestyle interventions. A review shows that eHealth technologies have been widely used in school settings to deal with multiple risk factors among students [17]. A systematic review of mobile health (mHealth) interventions for salt reduction identified more than 10 relevant intervention programs, which used short messaging services or other mHealth technologies to deliver salt reduction information to people [18].

Recent national data indicate that approximately 904 million Chinese have access to the internet [19]. This widespread access provides an opportunity for spreading health information via apps or other web-based tools. As yet, there has not been a salt reduction education program targeting primary school students using smartphone apps. We decided to take advantage of modern technologies to translate the successful experience of the School-EduSalt program and develop a more sustainable salt reduction program for broader scale-up in the future. Therefore, the AppSalt program was invented.

### AppSalt Program

The AppSalt program is an mHealth intervention program built upon the experience of the School-EduSalt program. In this program, a smartphone app named *AppSalt* will be designed to provide a platform for delivering standardized health education courses to grade 3 students (aged 8-9 years) and their parents. In addition to the app, we will also design and implement some offline activities. In total, there will be 5 intervention components in this program, aiming to mobilize children’s influence on family members to reduce the whole family’s salt intake. The intervention design is based on the health belief model [20] and the socioecological model [21]. The details of the 5 intervention strategies of the AppSalt program are described below:

- App-based health education on salt reduction: Health education courses on salt reduction will be delivered once every month through the app installed on a family member’s smartphone. Each lesson consisted of a 10-min video and a quiz to re-enforce important messages. Most of these lessons will have a practical session to help participants put what they have learned into practice. Health education lessons will cover essential knowledge and techniques for salt reduction, including the adverse effects of high salt intake, recommended salt intake amount, low-sodium salt, and skills in reducing salt used in cooking. The students and their family members can decide when and where to learn the lesson at their convenience. Parents will need to take the lessons with their children; students will be encouraged to share what they learned with their family members, especially the person responsible for home cooking. Through knowledge sharing between children and adults, the program aims to educate the whole family on salt reduction.
- Salt intake monitoring: The salt measurement module in the app can help participants monitor their salt intake and major salt contributors in their diet. This measurement consists of a 7-day food diary for the family, which requires the users to fill out on a daily basis and record the condiments used in home cooking, type and weight of processed food consumed at home, and frequency of eating out during the 7 days. After completing this 7-day diary, the app will calculate the average salt consumption using an embedded algorithm, which was proven to be highly consistent with salt intake measured by 24-hour urine collection in a previous validation study [22]. In addition, the app will automatically generate a salt reduction action plan for each family member according to their salt intake level and major sources of salt intake. In the action plan, we will suggest the participants try to reduce half of their salt intake. In addition, some practical skills for reducing salt intake will also be included in the action plan. The participants will be required to use the salt measurement module to monitor their progress in salt reduction every 3 months during the intervention period. Regular monitoring will inform the participants of their salt intake level and the gap between their current salt intake and the recommended amount.
- Competitions and awards: The AppSalt project team will design several school-based competitions and awards to motivate parents and children to engage in the app-based modules and enable interpersonal communication between students during their participation in these competitions. At each school, 4 competitions will be organized by the schoolteachers, including 1 art competition, 2 knowledge competitions, and 1 writing competition. After each competition, the top 30 students at each study site will be awarded certificates and prizes. The competitions will be organized once every 2 or 3 months.
- Parent meetings: Each intervention school will need to organize 3 to 4 parent meetings to encourage peer communication among the parents and to collect their feedback on the program. The parent meetings will be arranged at the beginning and end of each term. Each group meeting will have a specific topic related to recent courses or activities.
- Supportive environment: Posters will be provided to the schools to help create a supportive environment for salt reduction on campus. The themes of these posters will correspond to the topics of the health education videos delivered through the app. In addition, some practical tools for reducing salt intake will be provided to families in the intervention group, including salt-restriction spoons, salt containers, and reminding message stickers.

**Figure 1:**
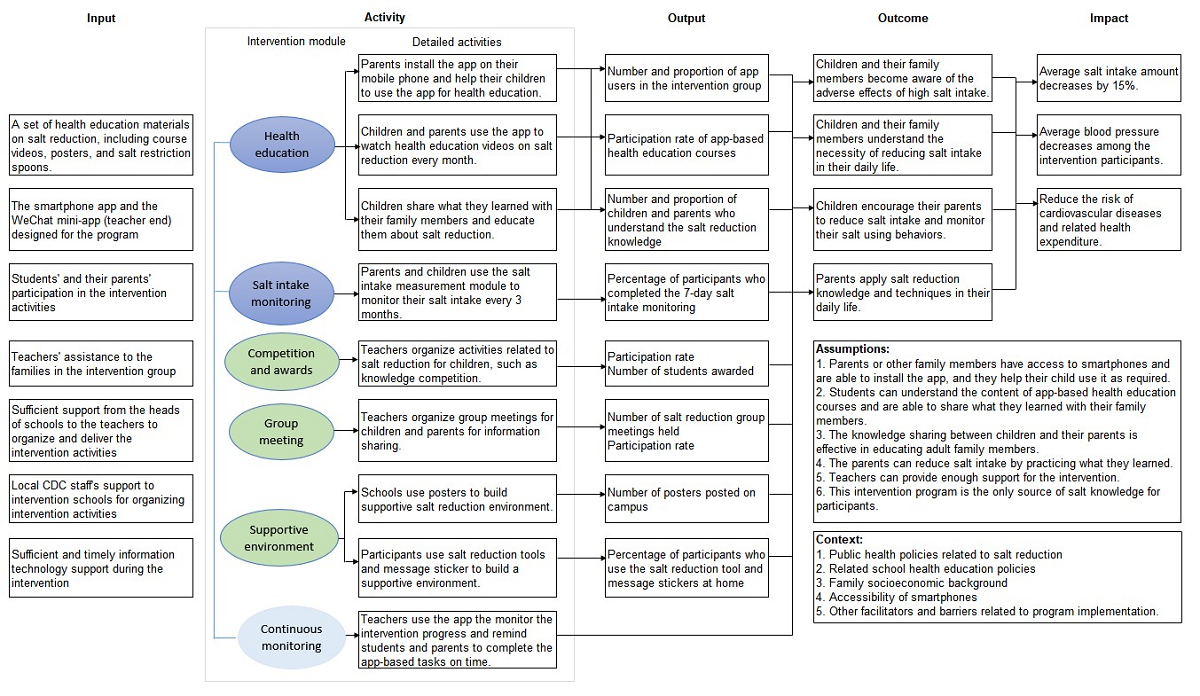
Logic model of the AppSalt program. CDC: Center for Disease Control and Prevention.

The logic model of the AppSalt program is shown in Figure 1, which outlines the inputs, intervention activities, intended outputs and outcomes, and long-term impacts. The central program office will manage the app and the web-based intervention activities in this program. The teachers in the intervention schools will deliver the offline intervention activities following the program office’s instructions. The teachers will be trained to keep activity logs of the offline intervention activities, which will be used as a data source for this process evaluation. We will develop a WeChat mini app (teacher end) for teachers to send messages to the families, check families’ progress, and ensure that all families complete the tasks in time.

### Effectiveness Evaluation

To test the effectiveness and cost-effectiveness of the AppSalt program, we will design a cluster randomized controlled trial and recruit 54 primary schools from 3 cities to participate in the trial. Half of the schools will be randomized to the intervention group and participate in the AppSalt intervention activities for 1 year. The other half will be the control group and continue with their usual health education curriculum. The primary outcome of the trial will be evaluated by the difference between the intervention and control groups in the change of salt intake measured by 24-hour urinary sodium from baseline to the end of the trial. The published protocol provides more details on the effectiveness evaluation of the AppSalt program [23]. This trial was registered at Chinese Clinical Trial Registry (ChiCTR1800017553).

### Process Evaluation and Objectives

The AppSalt program is the first multicomponent mHealth intervention program for salt reduction conducted at Chinese primary schools. The smartphone app used in this program will be an innovative tool for health education in the primary school context. Its acceptability and sustainability remain a question. In addition, multiple stakeholders will be involved in this program, including students, parents, teachers, school heads, and government officials. Their perceptions and experiences are valuable for refining the intervention design for future scale-up.

This process evaluation is designed to analyze the implementation of an mHealth tool in a real-world setting. Evaluating the implementation process will help researchers better understand contextual factors affecting the implementation, the feasibility of mHealth tools in real settings, and key stakeholders’ opinions on scaling up such a program in the future. Furthermore, this process evaluation will be helpful for other researchers and policy makers to replicate the AppSalt program and design their salt reduction program. The specific objectives of the AppSalt process evaluation are as follows:

- To evaluate the implementation of the AppSalt program in real-world settings, including fidelity, reach, dose delivered, and dose received
- To understand the underlying intervention mechanism of AppSalt program
- To identify the facilitators and barriers of implementing this program
- To collect stakeholders’ opinions and suggestions for scaling up the program.

## Methods

### Study Design

This process evaluation is informed by the theoretical framework proposed by Linnan and Steckler [24] and the UK Medical Research Council process evaluation guidance for complex interventions [25]. The fidelity of the implementation, intervention dose delivered and received, reach of the intervention strategies, and the contextual factors influencing the intervention will be evaluated. The definition of each evaluation component and data sources are listed in Table 1.

This process evaluation will be embedded in the full cluster randomized controlled trial of the AppSalt program. A mixed methods approach will be used to collect the process evaluation data of the AppSalt program throughout the intervention process. The major data sources and time of data collection are provided in Table 2.

**Table 1:** Process evaluation components and data collection methods.

Evaluation components	Definition	Data collection methods
Fidelity	To what extent is the intervention implemented as planned and carried out according to the principles of the intervention. Are there any adaptations made during the program?	App log and routine monitoring data
Dose delivered	Of the intended intervention strategies, how many were delivered and in what quantity?	App log, routine monitoring data, and interviews with teachers
Dose received	How satisfied are the teachers, children, and adults with the intervention? What is the extent to which children and adults engaged with and are receptive to the intervention?	Interviews with selected students, parents, and teachers
Reach	Of the intended participants, what percentage is reached by each strategy?	App log, routine monitoring data, and interviews with participants
Context	What are the barriers and facilitators to implementing each intervention strategy?	Interviews with local education and health authorities

**Table 2:** Time and methods of collecting process evaluation data.

Type of data			Time of collection
**Quantitative data**			
	App use logs	Automatically generated during the intervention process when the users log in to the app and use it for app-based intervention activities	
	Offline activity logs	Collected by the teachers when holding offline activities, including competitions and group meetings, during the intervention process	
	Routine monitoring data	Collected by a program coordinator every 2 weeks during the intervention process	
**Qualitative data**			
	Semistructured interviews with selected participants and key stakeholders	Within 1 month after completing the intervention	

### Data Collection

#### Quantitative Data Collection

The app use logs, offline activity logs, and routinely collected monitoring data will be the major quantitative data sources for process evaluation. The app use log will be automatically generated when the student or parent log in the app and use it for app-based intervention activities, including the time spent watching health education videos, marks gained from the quizzes, and frequency of 7-day salt intake monitoring. The app use log will be exported through the managing website by an authorized administrator after completing the 1-year intervention. The offline activity logs will be uploaded by teachers using the management tool (teacher end). We can extract detailed information about offline activities from these activity logs, including date, topics, and number of participants. Routine monitoring data will be collected every 2 weeks by a program coordinator during the intervention to evaluate the implementation of web-based courses and offline activities. If any problems identified were from the routine monitoring data, these problems will be provided to local collaborators for optimizing the intervention process. We will also monitor the dropout during the intervention and collect the reasons for dropping out.

#### Qualitative Data Collection

Qualitative data will be collected through semistructured interviews with participants and key informants to obtain more in-depth knowledge of their experience and feedback on the intervention [26]. Only the intervention group in the AppSalt program will be interviewed because of time and personnel constraints. In total, we plan to interview 27 students, 27 adult family members, 9 teachers, 9 school heads, and 9 representatives from local health and education authorities. The sample size of interviewees is based on similar process evaluation research [27,28] and might be increased if information saturation could not be reached.

**Figure 2:**
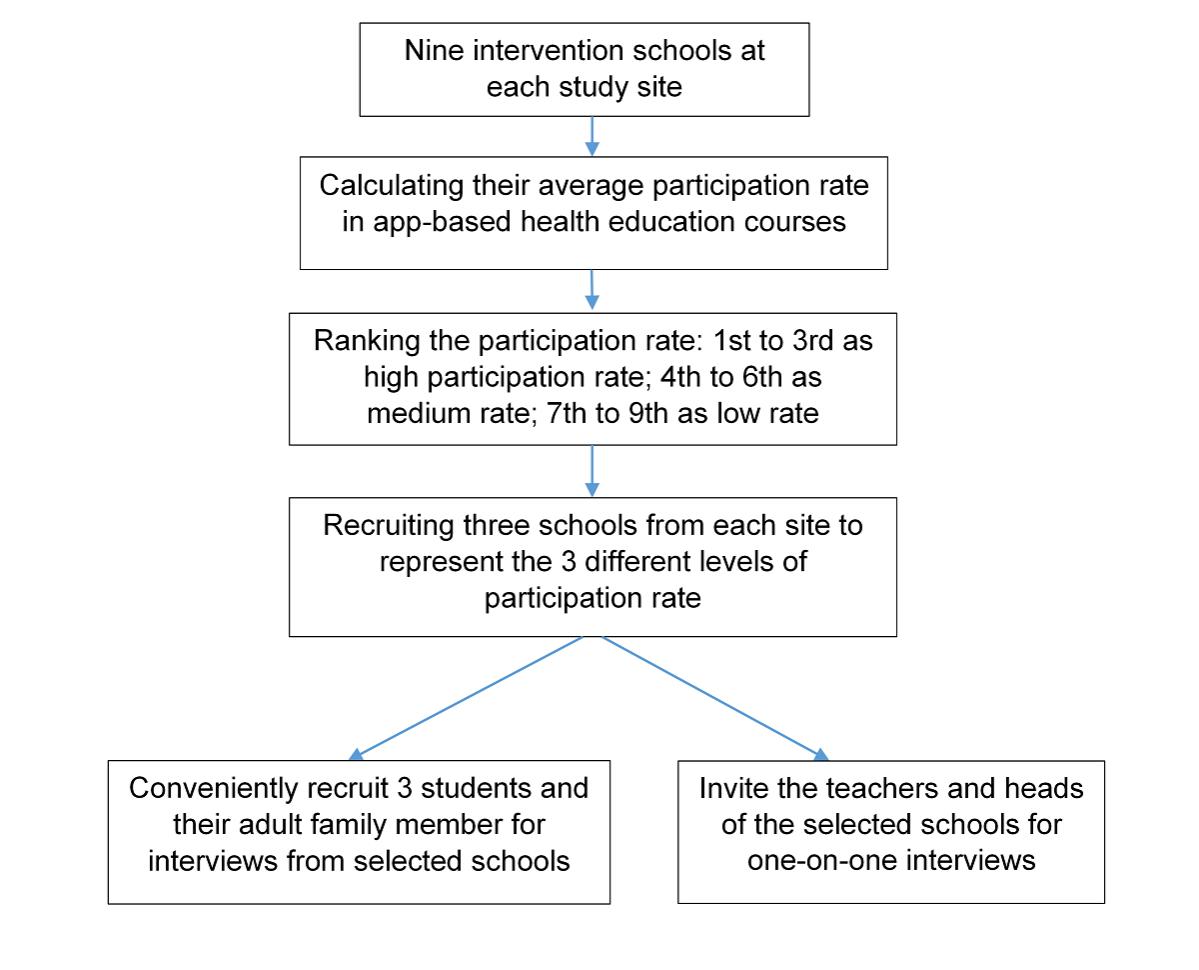
Process of selecting interviewees from intervention schools.

The maximum variation sampling method will be used for selecting intervention participants for interviews [29]. The process of choosing interviewees is shown in Figure 2.

A total of 3 intervention schools will be purposively selected from each site to represent different levels of implementation performance. The primary indicator of implementation performance will be the average participation rate of web-based health education courses, calculated from the app use log. Intervention participants, including students and their family members, will be conveniently recruited from the purposively selected intervention schools. The family member should be the person who installs the app on his or her smartphone. In addition, the teachers and school heads at each selected school will also be invited to participate in an individual interview.

For key stakeholders, we will interview local health and education authorities who are responsible for designing the primary school health education curriculum. The interviewees from these government departments will be nominated and invited by our local program partners. They will be interviewed for potential pathways of promoting salt reduction courses at primary schools for future scale-up. The interview guides are provided in Multimedia Appendix 1.

The interviews will be performed by trained interviewers who are experienced researchers from the George Institute and are not involved in the implementation of the AppSalt program. Key topics of the semistructured interviews will include the experience of intervention participation, difficulties encountered during their participation, suggestions for improvements, and transferability to other settings. All interviews will be audio recorded and transcribed verbatim for analysis. The transcripts will be checked for accuracy against the audio files, and corrections will be made as appropriate.

### Data Analysis

#### Quantitative Data Analysis

The participation rate of app-based health education courses and offline activities will be the key variable to represent participants’ adherence to the intervention program. The participation rate will be compared across sites. Descriptive statistics (comparisons of means, medians, or percentage as appropriate) will allow the research team to assess intervention delivery and provide information about the differential implementation rates of the 5 intervention components of the AppSalt program.

#### Qualitative Data Analysis

Qualitative data analysis will be performed in NVivo12 (QSR) using thematic analysis and following the step-by-step guide to increase its trustworthiness [30,31]. Initially, 2 researchers will individually code the first 10% (9/90) of the transcripts to familiarize themselves with the data and formulate their individual preliminary coding scheme, which should be generated deductively based on the process evaluation framework used to guide this study design [24]. The 2 coders will then discuss their coding schemes to reach a consensus before coding the remaining transcripts. The coding scheme will be discussed and refined iteratively during the coding process. After completing the initial coding of all transcripts, the researchers will search for themes from the patterns of codes. The themes will be generated using a deductive approach following the process evaluation components.

#### Data Triangulation

The qualitative data will be triangulated with the quantitative data during the interpretation phase to explain the 5 process evaluation dimensions. The preliminary plan for data triangulation is provided in Table 3. The quantitative data will evaluate how the implementation of the AppSalt program is achieved in different settings. The qualitative data will provide complementary information to the 5 evaluation components, which will allow researchers to investigate the in-depth reasons behind the varied implementation performance [32].

**Table 3:** Preliminary plan of quantitative and qualitative data triangulation.

Evaluation components	Quantitative indicators	Qualitative indicators
Fidelity	Frequency of intervention activities delivered and adaptation of intervention strategies in the process	Perceived level of implementation of key stakeholders, difficulties encountered in the implementation process, and parents’ and teachers’ feedback on the adaptations
Dose delivered	Number of app-based sessions, activities, and group meetings and intervention tools delivered to the families	Teachers’ experience and perception of the program and the difficulty of delivering the activities encountered by teachers and school heads
Dose received	Participation rate of intervention activities, length of viewing health education courses, and salt reduction knowledge received	Participants’ experience of the program, including satisfaction and perception of the program, and the most effective intervention module in participants’ opinions
Reach	Proportion of eligible students who participated in each intervention strategy, number of dropout, and retention rate	Reasons for participation and reasons for dropping out
Context	N/A^a^	Related contextual factors affecting the implementation of the program, including barriers and facilitators identified by multiple stakeholders

^a^N/A: not applicable.

## Results

This study was approved by the Peking University Institutional Review Board (No. IRB00001052-19096) and the Queen Mary Ethics of Research Committee (QMERC2020/22). The AppSalt program has completed its 1-year intervention in 27 selected schools at the end of 2019. The collection of quantitative process data was conducted alongside the intervention. Preliminary analysis of monitoring data was performed during the intervention to optimize the implementation process. Semistructured interviews of participants and key informants were conducted from October 2019 to December 2019. We interviewed 32 parents, 32 students, 9 teachers, 9 school heads, and 8 representatives from the local health and education authority. The audio recordings have been transcribed. Data analysis is currently underway. The results are expected to be published in 2021.

## Discussion

Process evaluation is becoming more recognized for its importance in evaluating program implementation and collecting evidence for better research translation [33,34]. This paper presents a mixed methods process evaluation protocol for the AppSalt program, an app-based program among primary school students and their families to translate the previous research evidence of the School-EduSalt program [15].

Salt reduction is challenging in China because of Chinese dietary habits, where the primary source of salt intake is home cooking [35]. The AppSalt program is designed to explore practical and sustainable intervention models for Chinese settings using modern technologies. This program is a complex salt reduction intervention program consisting of 5 intervention modules, which is innovative in its use of mHealth tools for salt reduction in school children and their families. In addition, the AppSalt program will be implemented at 3 study sites of different socioeconomic status. It would be unrealistic to expect perfect delivery of this complex intervention program across different contexts [36]. This process evaluation will help us investigate how this program will be delivered in different contexts and what level of implementation can be achieved by each intervention strategy using a mixed methods approach. The quantitative process data will be readily collected with the electronic intervention management system’s assistance during the intervention, which could well represent the real implementation situation [37]. For qualitative data, multiple stakeholders will be interviewed to provide in-depth feedback regarding implementing such a program in the real world [38]. These findings will help researchers refine the intervention design to a replicable model for scaling up.

Nevertheless, we acknowledge the limitations of this study because of some compromises made for time and resource constraints. First, only the intervention group in the AppSalt program will be interviewed. Therefore, potential contamination of the control group and other confounding factors during the intervention process might be neglected. Second, representatives from local health and education authorities will be invited for the interviews by our local research collaborators. This recruitment process might cause some recruitment bias. Third, the interviews will be performed after completing the 1-year intervention, which might cause some recall bias. To minimize potential recall bias, we will conduct all interviews within 1 month after the intervention.

Despite these limitations, this process evaluation of the AppSalt program can help researchers better understand the implementation of the AppSalt program in real-world settings and identify the barriers and facilitators of its implementation. This process evaluation will also make the causal mechanism of the AppSalt program explicit and will provide some experience of a lifestyle intervention for salt reduction in China and other countries.

## Appendix

Multimedia Appendix 1 Interview guides.

Multimedia Appendix 2 CONSORT-eHEALTH checklist (V 1.6.1).

## Abbreviations

BPUK: Blood Pressure UK
CASH: Consensus Action on Salt and Health
mHealth: mobile health
NCD: noncommunicable disease
NIHR: National Institute of Health Research
WASH: World Action on Salt and Health
