# Supplementary material for: App-Based Salt Reduction Intervention in School Children and Their Families (AppSalt) in China: Protocol for a Mixed Methods Process Evaluation
Source: JMIR Res Protoc. 2021 Feb 10;10(2):e19430. doi: 10.2196/19430 (PMC7904395; doi:10.2196/19430)
Supplement: Multimedia Appendix 1 [file resprot_v10i2e19430_app1.docx]

**Appendix 1**

**Process evaluation of an application-based salt reduction intervention in schoolchildren and their families (AppSalt) in China**

**Interview Guide**

**Introduction:** We are conducting a process evaluation of the AppSalt program. As part of this, we are conducting interviews with program participants, and key informants from schools and local health and education authorities. Here is the information sheet of this study. Please read it and ask me if you have any questions.

The topics of the interview are about your feelings and experiences related with your participation in the salt reduction program. We would also want to collect your suggestions for the promotion of the program in the future. Your experiences are very valuable for the researchers to understand the problems of program implementation and improve the program design.

Interviews will last approximately 40 minutes and your participation is voluntary – you may refuse to answer any question in the interview or stop the interview at any time. If you agree, we will record the interview for later analysis. The audio will be transcribed verbatim. All information being collected will be kept confidential. Personal identifying information is for the evaluation team only and will not be shared.

If you agree to participate, please sign your name on the consent form.

***Note to interviewers***: Please give the participant enough time to read the information sheet. Remember to give a copy of the signed consent form to the participant. The interview questions are different for different kinds of interviewees. Please find the right interview questions for this particular interviewee beforehand.

If the interviewee agrees to be recorded, please make sure to open to recorder. If the interviewee refuses to be recorded, please take as many notes of the interview as possible.

**Interview questions**

1. **For use with adult family members**

| **Topic 1 Overall participation in the intervention** | **Interviews notes** |
| --- | --- |
| 1. Are you busy? Do you have time to help your child with his/her study?   This is a warm-up question and can be changed according to the situation. |  |
| 1. What’s your motivation to participate in this salt reduction program? |  |
| 1. Can you list the activities that you and your family participated in during the intervention?   Four main activities that parents should take part:  □ Biweekly online health education courses and quizzes  □ Salt intake monitoring every three months  □ Salt reduction group meeting  □ Salt reduction competition activities |  |
| 1. Generally speaking, are you interest in this program?   No matter what the answer is, please ask the interviewee’s reasons of the answer. |  |
| **Topic 2 App related courses and tasks** |  |
| 1. This salt reduction program used the app to deliver health education courses. Is the teaching model suitable for your family?   Yes→ “Why is this model suitable for your family”  No→ “Why is it not suitable? What is the better way of health education for you and your family?” |  |
| 1. How do you usually use the app in your family? Parent alone? Student alone? Parent together with student?   Student alone→ “Would the student share the knowledge with you or other family members?” |  |
| 1. Have you encountered any difficulties when using the app?   Yes→ “If so, what are the impacts of these difficulties? Were these difficulties resolved?” |  |
| **Topic 3 Salt intake monitoring** |  |
| 1. In this program, there is a task called 7-day salt intake monitoring, which should be conducted every three months. Do you recall?   Yes→ Ask the following two questions.  No→ Open the app and help the interviewee to recall. If he/she stills answers no, please skip the following two questions. |  |
| 1. Is this 7-day salt intake monitoring task easy to complete?   Yes→ “Please describe how you completed the task at home.”  No→ “Why is it hard to do? What are the difficulties?” |  |
| 1. Do you think the 7-day salt intake monitoring is helpful for reducing salt intake? Why? |  |
| **Topic 4 Salt reduction group meeting** |  |
| 1. Have you ever taken part in the salt reduction group meeting at school?   Yes→ Proceed to the next question.  No→ “Have other family members taken part in the group meeting? Who?” |  |
| 1. If the interview has taken part in the group meeting：  - Do you remember what were discussed at the group meeting? - Do you think this meeting is interesting? - Is the group meeting helpful for reducing salt intake? |  |
| **Topic 5 Family salt reduction environment cultivation** |  |
| 1. In this program, we distributed some salt reduction leaflets and brochures. Have you received any of these materials?   Yes→ “Have you or your family members read these materials? Are these materials helpful?”  Not sure or No→Show the materials to the interview and help him/her recall. |  |
| 1. At the beginning of the program, we offered salt restriction spoons and small salt container to each family. Is your family using these tools now?   Yes→ “Are these tools helpful for reducing salt intake? And why?”  No→ “What are the reasons?” |  |
| **Topic 6 Family salt reduction practices** |  |
| 1. We have talked about the courses and activities that you and your child participated in during the past year. Which one is the most effective and important activity for you and your family reducing salt intake?   If possible, please ask the interviewee to rank the intervention activities according to their importance for salt reduction. |  |
| 1. Do you support reducing salt intake?   No→ “Why？”  Yes→ “Do you think it is difficult to reduce salt intake? What are the main difficulties?” |  |
| 1. Do other family members support reducing salt intake?  - Who is the most active supporter for salt reduction in your family? And why? Could you please give some examples? - Are there any family members against salt reduction? And why? |  |
| **Topic 7 Overall suggestions** |  |
| 1. What is good about this project? What is not good about this project? How can it be improved? |  |
| 1. If we are going to scale up this program in all primary schools in this city, do you have any suggestions? |  |
| 1. Anything else you would like to add? |  |

**2. For use with students**

| **Topic1 Overall participation in the intervention** | **Interview notes** |
| --- | --- |
| 1. Can you list the activities that you and your family participated in during the intervention?   Four main activities that the family should take part:  □ Biweekly online health education courses and quizzes  □ Salt intake monitoring every three months  □ Salt reduction group meeting  □ Salt reduction competition activities |  |
| 1. Generally speaking, are you interest in this program?   No matter what the answer is, please ask the interviewee’s reasons of the answer. |  |
| **Topic 2 App related courses and tasks** |  |
| 1. Whose mobile phone is the AppSalt app installed on? Please describe how do you use the app with your family member? Do you use it alone? Or together with your family member? |  |
| 1. What knowledge on salt have you learned through the program? |  |
| 1. What techniques for reducing salt have you learned through the program? |  |
| 1. Do you like to use the AppSalt app to watch videos and learn health knowledge? Or do you prefer to listen to teacher’s lecture in the classroom? Why? |  |
| 1. Do you think the app is easy to use? Why?   No matter what the answer is, please ask the interviewee’s reasons of the answer. |  |
| **Topic 3 Salt intake monitoring** |  |
| 1. How do you and your parents perform the 7-day salt intake monitoring? Do you take part in this activity? |  |
| 1. Can you perform salt intake monitoring without your parent’s help? |  |
| **Topic 4 Offline salt reduction activities** |  |
| 1. Have you taken part in the salt reduction group meeting at school?    - Yes→ Please proceed with the following two questions. 2. Do you think it is interesting? 3. Is it helpful for reducing family salt intake? Why?    - No→ “Why?” |  |
| 1. Have you taken part in the salt reduction artistic competition or knowledge competitions?   Yes→ Please proceed with the following questions：   1. Do you think these competitions are interesting? Are these activities helpful for reducing salt intake? Why? 2. What are your motivations for taking part in these activities? 3. Have you encountered any difficulties during your participation in these activities?   No→ Please ask the reasons of not taking part. What kind of activities would be more attractive to you? |  |
| **Topic 5 Family and campus salt reduction environment cultivation** |  |
| 1. Have you ever noticed the salt reduction posters on campus or in the classroom?   Yes→ Please proceed with the following questions：   - 1. Can you tell me the contents of these posters?   2. Are these posters helpful for you to learn salt reduction knowledge? |  |
| 1. Have you taken the salt reduction leaflets and brochures to your family members? Did they read these materials? Are these materials helpful for you and your family members to learn knowledge on salt?   Please show the photos of these materials to the interviewee and help him/her to recall. |  |
| 1. At the beginning of the program, we offered salt restriction spoons and small salt container to each family. Is your family using these tools now?   Please show the photos of these tools to the interviewee and help him/her recall.  Yes→ “Are these tools helpful for reducing salt intake? And why?”  No→ “What are the reasons?” |  |
| **Topic 6 Salt reduction knowledge sharing within the family** |  |
| 1. Who usually cooks for you at home? |  |
| 2. Have you ever shared the knowledge on salt with this family cook?   - Yes→ Please proceed with the following questions：  1. Whom did you tell? How did you tell him/her? 2. Did they listen to you? Did they reduce salt for cooking after you told him/her?  - No→ Please ask “Why not telling them?” |  |
| **Topic 7 Family salt reduction practices** |  |
| 1. Have you ever asked your parent to reduce the salt used for cooking?  - Yes→ Please proceed with the following questions：   1. How did you do that? Please give some examples.   2. What are the difficulties? Did they listen to you? - No→ Please ask “Why not?” |  |
| 1. Do your family members support reducing salt intake?  - Who is the most active supporter for salt reduction in your family? And why? Could you please give some examples? - Are there any family members against salt reduction? And why? |  |
| **Topic 8 Overall suggestions** |  |
| 1. We have talked about the courses and activities that you participated in during the past year. Which one is the most effective and important activity for you and your family reducing salt intake?   If possible, please ask the interviewee to rank the intervention activities according to their importance for salt reduction. |  |
| 1. What is good about this project? What is not good about this project? How can it be improved? |  |
| 1. If you are asked to tell one sentence to help other students reduce salt intake, what would you tell them? |  |
| 1. Anything else you would like to add? |  |

1. **For use with teachers**

| **Topic 1 Overall participation in the intervention** | **Interview notes** |
| --- | --- |
| 1. Please briefly introduce the implementation of AppSalt program in your class. |  |
| 1. According to your understanding, are the students and parents interested in this program? |  |
| **Topic 2 Courses and tasks in this program** |  |
| 1. What is the biggest difficulty when you ask the students and parents to complete the tasks? How did you deal with these difficulties? |  |
| 1. Have you ever used any methods to increase the participation rate of the salt reduction activities?   Yes→ “Could you please give more details? Did these methods work?” |  |
| 1. How should we improve the courses in this program if we are going to promote this intervention? Contents and format? |  |
| 1. In this program, there is a task call 7-day salt intake monitoring, which is scheduled every three months. Do you think this task is hard to do for the families? If so, what are the difficulties?  - What are the effects of this task on the implementation of the whole project? Benefits or harm? - Do you have any suggestions regarding promoting the 7-day salt intake monitoring module in the future? |  |
| 1. Generally speaking, is the course frequency and workload suitable for the students and their families?   No→ “How should we adjust it?” |  |
| 1. Is the model of teaching through app acceptable for the students and their parents? Why?   Not acceptable→ “What is the better way of delivering salt reduction courses in primary schools?” |  |
| **Topic 3 Offline salt reduction activities** |  |
| 1. During the past year, several salt reduction activities were organized. What is overall all participation rate of these activities? Were students and parents active? How would you evaluate the outcome of these activities? |  |
| 1. Have you encountered any difficulties when organizing these activities?   Yes→ “Did you take any measures to due with these difficulties? Did it work?” |  |
| 1. In addition to these activities, is there any other activities that would be better? |  |
| 1. In addition to the activities organized by the project office, have you organized any other activities related with salt reduction?   Yes→ “What activity was organized? What is the outcome?” |  |
| **Topic 4 Salt reduction group meetings** |  |
| 1. During the intervention, several groups were organized. Did you encounter any difficulty when organizing these meetings? |  |
| 1. Are these group meeting helpful for the project implementation? Why? |  |
| 1. Do you have suggestions regarding the arrangement of group meetings? Contents? Frequency? |  |
| **Topic 5 Campus salt reduction environment cultivation** |  |
| 1. The posters were designed for cultivating salt reduction environment on campus. Did you have any difficulties of using these posters?   Yes→ How did you deal with these difficulties? |  |
| 1. Are these posters helpful for cultivating supportive salt reduction environment on campus?   Not helpful→ Are there better way of cultivating supportive environment? |  |
| **Topic 6 Overall suggestions** |  |
| 1. Do you support reducing salt intake?   Yes→ “What are your motivations?”  No→ “What are your reasons against salt reduction?” |  |
| 1. What is good about this project? What is not good about this project? How can it be improved? |  |
| 1. Are you overburdened in this program? From the perspective of teachers, what kind of support and motivations are helpful for promoting this program in the future? |  |
| 1. If the program is conducted at another school, what will you tell the teach to better implement the program? |  |
| 1. For scaling up, is it possible to incorporate this program in the usual health education curriculum?   No→ “Why? What are the barriers?”  Yes→ “If we are going to do that, do you have any suggestions?” |  |
| 1. Do you have any other suggestions for scaling up this program in the future? |  |
| 1. Anything else you would like to add? |  |

**4. For use with school heads**

| **Interview questions** | **Interview notes** |
| --- | --- |
| 1. Please briefly introduce the implementation of AppSalt program in your class. |  |
| 1. This program cannot be implemented properly without school’s support. During the past year, what kind of support has been provided to the students and teachers for implementing this program? What are the effects of these measures? |  |
| 1. Did you and the teachers encounter any difficulties for organizing salt reduction activities? Could you please describe the difficulties and your ways of dealing with these difficulties? |  |
| 1. In the AppSalt program, the app is a main intervention platform which delivers health education videos and other activities. How do you evaluate the effectiveness of using app for teaching health knowledge from the point of a school manager? What are the strengths and weaknesses of this tool? |  |
| 1. From the perspective of project implementation, what is good about this project? What is not good about it? How can it be improved? |  |
| 1. Are there any facilitators for implementing salt reduction program in the school or in the society? |  |
| 1. Do you have any suggestions for other head-teachers if their schools are going to implement a similar program? |  |
| 1. For scaling up, is it possible to incorporate this program in the usual health education curriculum?   No→ “Why? What are the barriers?”  Yes→ “If we are going to do that, do you have any suggestions?” |  |
| 1. Do you have any other suggestions for scaling up this program in the future? |  |

**5. For use with CDC representatives**

| **Interview questions** | **Interview notes** |
| --- | --- |
| 1. Please briefly introduce the implementation of AppSalt program in the 9 intervention schools in this city. |  |
| 1. Please introduce the role of CDC in implementing the AppSalt program. What are your tasks? |  |
| 1. During the past year, how did CDC collaborate with local education department? What’s the impact of these collaboration for implementing this program? |  |
| 1. Generally speaking, is the workload and arrangement of AppSalt program reasonable? Should the project office make any adjustment? How should it be adjusted? |  |
| 1. During implementing this program, what difficulties have you and your team encounter? And how do you deal with these difficulties? |  |
| 1. From the perspective of overall design and implementation, what is good about this project? What is not good about it? How can it be improved? |  |
| 1. Are there any facilitators for implementing salt reduction program in the school or in the society? |  |
| 1. For scaling up, is it possible to incorporate this program in the usual health education curriculum?   No→ “Why? What are the barriers?”  Yes→ “If we are going to do that, do you have any suggestions?” |  |
| 1. For scaling up the AppSalt program, how can we better utilize the CDC’s function? How to use CDC’s resources for better implementing the salt reduction program in schools? |  |
| 1. Do you have any suggestions, if we are going to promote this program in the third Grade of all primary schools? |  |
| 1. Do you have any suggestions for other CDC staffs if they are going to implement a similar program in their city? |  |
| 1. Anything else you would like to add? |  |

**6. For use with education department representative**

| **Interview questions** | **Interview notes** |
| --- | --- |
| 1. Please describe the situation of health education in the primary schools of this city? Is there a health education curriculum? If so, what’s the frequency of health education course? Are there specialized health education teachers? |  |
| 1. How do you evaluate the AppSalt program? |  |
| 1. What kind of support did the education authority provide for implementing this program during the past year? |  |
| 1. Are there any facilitators for implementing salt reduction program considering the resources and conditions of this city? |  |
| 1. Are there any barriers for implementing salt reduction program considering the resources and conditions of this city? |  |
| 1. From the perspective of overall design and implementation, what is good about this project? |  |
| 1. From the perspective of overall design and implementation, what is not good about this project? How can it be improved? |  |
| 1. For scaling up, is it possible to incorporate this program in the usual health education curriculum?   No→ “Why? What are the barriers?”  Yes→ “If we are going to do that, do you have any suggestions?” |  |
| 1. Do you have any suggestions for the education departments in other cities if they are going to implement a similar program in their city? |  |
| 1. Do you have any suggestions, if we are going to promote this program in the third Grade of all primary schools? |  |
| 1. Anything else you would like to add? |  |

**7. For use with health department representatives**

| **Interview questions** | **Interview notes** |
| --- | --- |
| 1. How do you evaluate the AppSalt program? |  |
| 1. What kind of support did the health authority provide for implementing this program during the past year? |  |
| 1. From the perspective of overall design and implementation, what is good about this project? |  |
| 1. From the perspective of overall design and implementation, what is not good about this project? How can it be improved? |  |
| 1. Are there any other salt reduction programs being implemented during the past year? If so, what are the impacts of these programs? |  |
| 1. The government has issued a few policies related with salt reduction recently, such as the *Healthy China Initiative*. What is the impact of these policies for implementing salt reduction programs in this city? |  |
| 1. Are there any barriers for implementing salt reduction program considering the resources and conditions of this city? |  |
| 1. Do you have any suggestions, if we are going to promote this program in the third Grade of all primary schools? |  |
| 1. Anything else you would like to add? |  |
